# Automated cell count in body fluids: a review

**DOI:** 10.1515/almed-2021-0011

**Published:** 2021-03-15

**Authors:** María José Alcaide Martín, Laura Altimira Queral, Laura Sahuquillo Frías, Laura Valiña Amado, Anna Merino, Luis García de Guadiana-Romualdo

**Affiliations:** Committee of Laboratory Tests, Spanish Society of Laboratory Medicine (SEQC-ML), Barcelona, Spain; Hematologic Biology Committee, Spanish Society of Laboratory Medicine (SEQC-ML), Barcelona, Spain

**Keywords:** automated cell count, body fluids, chamber count, optical microscopy

## Abstract

Body fluid cell counting provides valuable information for the diagnosis and treatment of a variety of conditions. Chamber cell count and cellularity analysis by optical microscopy are considered the gold-standard method for cell counting. However, this method has a long turnaround time and limited reproducibility, and requires highly-trained personnel. In the recent decades, specific modes have been developed for the analysis of body fluids. These modes, which perform automated cell counting, are incorporated into hemocytometers and urine analyzers. These innovations have been rapidly incorporated into routine laboratory practice. At present, a variety of analyzers are available that enable automated cell counting for body fluids. Nevertheless, these analyzers have some limitations and can only be operated by highly-qualified laboratory professionals. In this review, we provide an overview of the most relevant automated cell counters currently available for body fluids, the interpretation of the parameters measured by these analyzers, their main analytical features, and the role of optical microscopy as automated cell counters gain ground.

## Introduction

Body fluid testing is an important part of the workload in clinical laboratories [1]. Testing includes the analysis of biochemical, cytological and microbiological tests that grouped are used for the diagnosis of diseases causing accumulation and/or alterations in their composition.

The gold-standard for the cytologic analysis of body fluids is manual cell counting by expert laboratory professionals using a count chamber and evaluation of the differential count by optical microscopy after cytocentrifugation and staining of the specimen [2]. This method has some limitations, namely: staining conditions vary across laboratories, which affects quality. This technique is time-consuming, which increases intra-laboratory turnaround time; inter- and intra-observer variability results in high imprecision, and the equipment requires operation by highly-qualified personnel. Additionally, cytocentrifugation may affect cell recovery, thereby resulting in cell loss or morphological changes, which may yield misleading results [3]. Therefore, potential sources of error require that the results obtained by microscopy are interpreted with caution [4].

To partially overcome these limitations, the *in vitro* diagnostics industry has adapted their blood and urine analyzers to enable the analysis of extravascular body fluids (EBF). This has simplified EBF analysis, improved cell count precision, and contributed to cell count harmonization [5].

However, these analyzers can only be operated by laboratory professionals who are thoroughly familiar with the characteristics and limitations of these systems. Indeed, their limitations sometimes make microscopic review necessary. Therefore, laboratory professionals must previously verify the specifications of the analyzers for these specimens [3], [6].

## Materials and methods

### Search strategy and inclusion criteria

A literature search was performed on Pub Med without time or language restrictions. The search terms body fluid AND cell count AND (automated OR automation) AND/OR microscopy were used to search for studies comparing automated cell counting *vs*. cell counting chamber, where differential cell counting was also performed by the gold-standard method, optical microscopy. We also searched for grey literature on Google.

During the selection process, all documents retrieved were reviewed independently by two investigators. Titles and abstracts were reviewed to detect and exclude unrelated studies, whereas the texts found to be potentially eligible underwent full-text reading. Differing opinions were discussed and solved with the intervention of a third reviewer, when necessary.

## Results

### Automated cell counts for body fluids

The new counters incorporated into blood and urine analyzers have proven to be useful for cell counting for other body fluids. These counters provide determination of the following analytes:

– Total nucleated cell count (TNC-BF), term recommended by the *Clinical and Laboratory Standards Institute* (CLSI) [7].
– Total white blood cell count (WBC-BF).
– Red blood cell count (RBC-BF).
– Polymorphonuclear cell count (PMN-BF) and mononuclear cell count (MN-BF), expressed as percentages and absolute values.
– For research purposes, some analyzers measure other leukocyte populations or report the presence of other types of cells.

### Hematology analyzers

#### Sysmex analyzers (*Roche Diagnostics*)

Some series of Sysmex hematology analyzers, marketed in Spain by Roche Diagnostics, contain a dedicated mode (“XN-BF *mode*”) approved by the *Food and Drug Administration* (FDA) that performs cell counts for body fluids. This mode uses fluorescence flow cytometry for TNC-BF and WBC-BF and impedance flow cytometry for RBC-BF. The XN and XN-L series became available in the market in 2011 and 2015, respectively. However, the XT and XE series continue to be used in numerous studies, as they are still available in many laboratories [8], [9], [10]. Apart from performing TNC-BF, WBC-BF and RBC-BF count, they also analyze PMN-BF and MN-BF as research parameters. The latter are defined as parameters assayed by the analyzer but not validated for clinical use, including, but not limited to neutrophils (NE-BF), eosinophils (EO-BF), lymphocytes (LY-BF), monocytes (MO-BF) and high-fluorescence cells (HF-cells), having higher-sensitivity for RBC-BF. These analyzers reduce turnaround time, only require a small volume of specimen (88 μL for the XN series and 70 μL for the XN-L series); provide quality control material for the mode of body fluids; do not require specimen preparation except for synovial fluid [11]; have a flagging system for the presence of atypical cells, which is useful for indication of microscopy review; have an adequate limit of quantification for low-cellularity fluids such as CSF; and yield a differential count for four populations, which transferability has been recently evaluated [12].

The utility of the XN and XN-L series as a practical and reliable alternative to manual counting has been demonstrated in several studies [12], [13], [14], [15], [16], [17]. Of note, a tendency to overestimation has been observed in some cell populations [13], [15]. However, these differences are not clinically relevant, given the high level of concordance (95%) between the two methods in white blood cell count for body fluid classification [15]. Indeed, the level of concordance between the two methods increases in low-cellularity specimens [8], [15]. A recent study assessing the utility of these analyzers to assess cellularity in pericardial fluid from cardiac surgery patients confirmed its efficacy for total cell counting, but question their utility differential count due to the lack of transferability of results. This is credited to the particular composition of pericardial fluid, as compared to other serous fluids, with a high percentage of mesothelial cells, which trend to aggregate. Therefore, the authors identify optical microscopy as the most reliable method for the analysis of pericardial fluid [18].

The reported imprecision of the XN series for WBC-BF, TNC-BF, PMN-BF and MN-BF at low counts (<10 cells/μL) in CSF is below 20%, as recommended for this type of assay [19], [20], and below 10% for higher counts [11], [15]. Using specific control materials for body fluids, imprecision for the two levels is below 10% for WBC-BF, PMN-BF and MN-BF [13], which is consistent with the imprecision reported for XN-L analyzers [21]. Table 1 shows other analytical specifications of automated counters.

**Table 1:** Analytical characteristics of hematology analyzers and GLOCyte for body fluid testing.

References	Fluids	LOB	LOD	LOQ	Carryover	Linearity	
		(as cells/μL)					
**Sysmex XN series analyzers**							

[8]	CSF, ascitic, pleural and peritoneal dialysis fluid	Not assessed	Not assessed	WBC-BF: 5.0	<0.05%	WBC-BF r^d^=0.99 (mean bias in the low count range [5–12/μL]: −22.68% with a mean recovery of 81.8%)	
						RBC-BF r^d^=0.99 (AsF differences between both counts were higher in range of 0–200/μL range)^a^	
[11]	Synovial fluid	TNC-BF: 0.9	TNC-BF: 2.0	TNC-BF: 2.6	0.00%	TNC-BF: r=1.0 (range: 43–46,718/μL)	
		WBC-BF: 0.6	WBC-BF: 1.6	WBC-BF: 2.9		WBC-BF: r=1.0 (range: 43–46,688/μL)	
		PMN-BF: 0.6	PMN-BF: 1.6	PMN-BF: 22.7		PMN-BF: r=1.0 (range: 41–40,216/μL)	
		MN-BF: 0.0	MN-BF: 1.1	MN-BF: 9.1		MN-BF: r=1.0 (range: 2–6,854/μL)	
[15]	CSF	TNC-BF: 0.7	TNC-BF: 1.6	TNC-BF: 3.0	<0.1%	TNC-BF r=1^b^	
		WBC-BF: 0.3	WBC-BF: 1.2	WBC-BF: 3.0		WBC-BF r=1^b^	
		MN-BF: 0.2	MN-BF: 1.6	MN-BF: 6.0			
		PMN-BF: 0.3	PMN-BF: 1.3	PMN-BF: 8.0			

**Sysmex serie XN-L analyzers**							

[17]	CSF, ascitic, pleural and others (including peritoneal dialysis fluid, pericardial and synovial fluid)		CSF:	CSF:	<0.06%	CSF:	
		RBC-BF: 0	RBC-BF: 200	RBC-BF: 6,100		RBC-BF: 1,000–4,800	R^d^=1
		TNC-BF: 0	TNC-BF: 1.0	TNC-BF: 5.4		TNC-BF: 9–4,147	
		PMN-BF: 0	PMN-BF: 1.4	PMN-BF: 8.2		PMN-BF: 7–3,312	
		MN-BF: 0	MN-BF: 0.9	MN-BF: 3.9		MN-BF: 9–1,203	
			LPl:	LPl:		LPl:	
			RBC-BF: 500	RBC-BF: 1,900		RBC-BF: 100–7,900	R^d^=1
			TNC-BF: 1.8	TNC-BF: 8.9		TNC-BF: 8–4,191	
			PMN-BF: 1.0	PMN-BF: 8.0		PMN-BF: 9–3,304	
			MN-BF: 1.3	MN-BF: 10.6		MN-BF: 10–2,457	
			AsF:	AsF:		AsF:	
			RBC-BF: 400	RBC-BF: 2,400		RBC-BF: 2,000–8,100	R^d^=1
			TNC-BF: 1.0	TNC-BF: 3.5		TNC-BF: 5–5,571	
			PMN-BF: 0.8	PMN-BF: 8.7		PMN-BF: 9–3,103	
			MN-BF: 1.2	MN-BF: 9.0		MN-BF: 12–1,753	
**BC-6800 (Mindray) analyzer**							

[30]^c^	Ascitic, pleural and peritoneal dialysis fluid	WBC-BF: 3.0	WBC-BF: 8.0	WBC-BF: 8.0	<0.05%	Not assessed	
[31]^c^	CSF	TNC-BF: 0.0	TNC-BF: 3.0	TNC-BF: 4.0	<0.3%	TNC-BF: 4–1,902/μL; r^d^=1.00	
		WBC-BF: 0.0	WBC-BF: 3.0	WBC-BF: 6.0		WBC-BF: 4–1,902/μL; r^d^=1.00	
[32]^c^	Ascitic and pleural fluid	TNC-BF: 1.0	TNC-BF: 3.0	TNC-BF: 4.0	0.00%	TNC-BF: 8–3,965/μL; r^d^=0.99	
		WBC-BF: 1.0	WBC-BF: 3.0	WBC-BF: 3.0		WBC-BF: 8–3,936/μL; r^d^=0.99	
				PMN-BF: 22.0		PMN-BF: 24–3,063/μL; r^d^=0.99	
				MN-BF: 12.0		MN-BF: 18–2,279/μL; r^d^=0.99	
[35]^c^	Synovial fluid	TNC-BF: 6.0	TNC-BF: 15.0	TNC-BF: 15.0	< 0.3%	TNC-BF: 42–29,234/μL; r=0.97	
		WBC-BF: 6.0	WBC-BF: 16.0	WBC-BF: 16.0		WBC-BF: 42–29,221/μL. r=0.96	
				PMN-BF: 16.0		PMN-BF: 40–25,421/μL; r=0.98	
				MN-BF: 23.0		MN-BF: 2–3,975/μL; r=0.92	

S**erie Cell-Dyn Sapphire analyzer (Abbott Diagnostics)**							

[38]	CSF, ascitic, pleural and peritoneal dialysis fluid	WBC-BF: 2.3	Not assessed	WBC-BF: 20	0.16% ^d, e^	WBC-BF: 5–900/μL; r^d^=1	
		RBC-BF: 0		RBC-BF: 3,000		RBC-BF: 3,000–90,000/μL; r^d^=1	

**Unicel DxH 800 analyzer (Beckman Coulter)**							

[21]	CSF, pleural, ascitic, synovial, bronchoalveolar lavage and peritoneal dialysis fluid	TNC-BF: 12	TNC-BF: 18	TNC-BF: 37	Not assessed^d^	TNC-BF: 20–89,000/μL^e^	
		RBC-BF: < 1000	RBC-BF: < 1000	RBC-BF: > 5000		RBC-BF: 1,000–6,200,000/μL^e^	

**GLO**C**yte analyzer (Advanced Instruments Inc.)**							

[44]	CSF	TNC-BF: 0.47	TNC-BF: 1.2	TNC-BF: 2.6	Not assessed	Not assessed	
		RBC-BF: 0.73	RBC-BF: 0.8	RBC-BF: 2.0			
[45]		TNC-BF: <1	TNC-BF: 1	TNC-BF: 3		Analytic measurement range	
		RBC-BF: <1	RBC-BF: 1	RBC-BF: 2		TNC-BF: 3–123/μL	
						RBC-BF: 2–123/μL	

LOB, limit of blank; LOD, limit of detection; LOQ, limit of quantification (minimum cell count that can be obtained with a coefficient of variation ≤ 20%) ; PeritF, peritoneal dialysis fluid; CSF, cerebrospinal fluid; TNC-BF, total cell count; WBC-BF, leukocyte count; PMN-BF, polymorphonuclear cell count; MN-BF, mononuclear cell count; RBC-BF, total RBC count. ^a^ Analytical sensitivity (provided by the manufacturer): 1,000/μL. ^b^ Count range in tests where linearity was assessed (for WBC-BF: 6–785 in lab 1 and 1–640 in lab 2, and for TNC-BF: 6–787 in lab 1 and 1–654 in lab 2). ^c^ Data on characteristic AsF in RBC not available (RBC-BF). ^d^ The manufacturer recommends that a rinse cycle with a dilutant is performed prior to specimen processing. ^e^ Data provided by the manufacturer.

Experience with these devices has generated a considerable body of knowledge on potential interfering factors in cell counting. Thus, the presence of yeasts may interfere with WBC-BF, TNC-BF and HF-BF and generate a characteristic pattern on the scattergrams (“*blue surfboard pattern*”) [22]. Hence the relevance of routine reviews. Likewise, false increases of WBC-BF have been observed in CSF of oncologic patients treated with *Depocyte*, a chemotherapy agent (cytarabine) used for the treatment of neoplastic meningitis. In this case, this type of specimen cannot be analyzed by automated cell counting [23].

#### Advia analyzers (*Siemens Healthineers*)

Advia 2120/2120i incorporates a *Unified Fluids Circuit* for CSF analysis. This technology performs RBC-BF, WBC-BF and PMN-BF, MN-BF, NEU-BF, LY-BF and MO-BF counting, expressed as absolute values and percentages. It also includes an FDA-approved specific application for body fluids that measures TNC-BF and RBC-BF in pleural, ascitic, peritoneal and synovial fluid previously treated with hyaluronidase [24].

According to FDA reports, the limit of detection of these analyzers is adequate for serous fluid processing, with an imprecision <20% even at low-cellularity values, with a rate of carryover contamination <0.1%. Linearity studies confirm that deviations are <10% in the ranges studied. In addition, there is specific quality control material available for body fluids. However, these analyzers have some limitations.

– CSF analysis requires previous specimen dilution and preparation, which increases laboratory turnaround time.
– Although transferability studies confirm this analyzer as an alternative to manual counting in ascitic and pleural fluid [24], [25], [26], it has not been confirmed for peritoneal dialysis fluids [27]. In addition, a low correlation in RBC-BF count has been observed between this automated analyzer and flow cytometry, used as the reference technique (r=0.545) [28].
– This counter is not equipped with a flagging mechanism for the presence of atypical cells.
– Misleading results for leukocyte count have been obtained in CSF with RBC-BF above 1500/μL [28] and 250/μL [29].

#### BC-6800 BF analyzer (*Mindray Medical International*)

**Figure 1:**
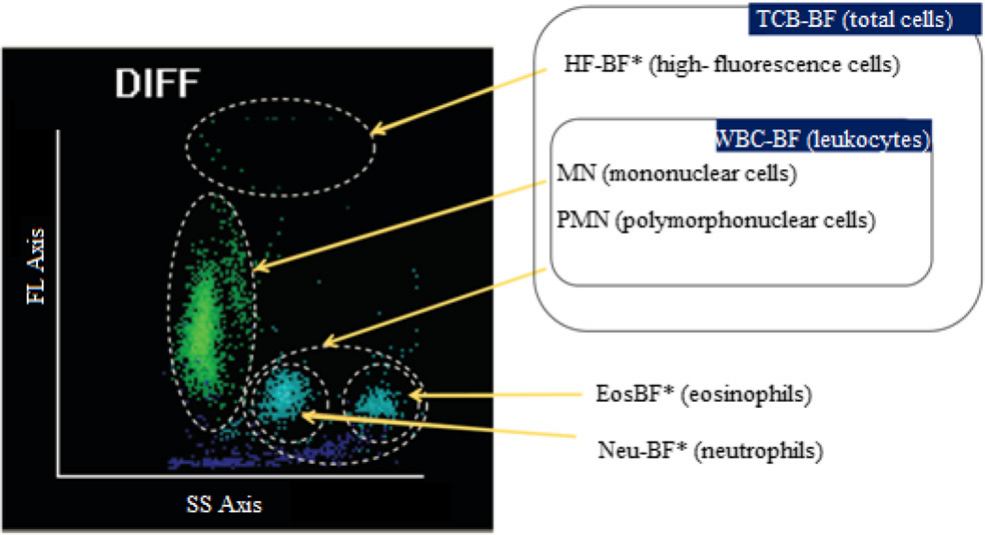
Characteristic tridimensional scattergram for differentiating (DIFF) nucleated cells in the mode for body fluids (BF mode) of the BC-6800 analyzer. Cells are grouped according to their morphological complexity (SS axis), size (FS axis) and nucleic acid content (FL axis). Adapted from https://www.mindray.com/en/product/BC-6800.html.

BC-6800 is a blood analyzer incorporating a specific mode that has not yet been approved by the FDA for cell count in CSF, synovial and serous fluids. Its utility in peritoneal dialysis fluids has also been recently assessed [30]. This analyzer performs TNC-BF, WBC-BF, MN-BF and PMN-BF determination and reports HF-cell (HF-BF*), neutrophil (Neu-BF*) and eosinophil count (Eos-BF*) as research parameters. The distribution of cellularity can be observed on a scattergram (Figure 1). This mode measures nucleated cell count by hydrodynamic focusing flow cytometry (SF Cube) after lysis and fluorescent staining of nucleated cells. These cells are classified on a 3D scattergram according to their internal complexity, size and nucleic acid content. Additionally, this analyzer performs impedance flow cytometry of RBC-BF in body fluids.

The main advantages of this analyzer are the availability of internal quality control material, its short turnaround time (<3 min), the low volume of sample required (150 μL) and that the specimen does not require pretreatment.

Recent studies assessing its analytical characteristics (Table 1) and the transferability of results with respect to the method of reference [25], [30], [31], [32], [33], [34], [35] confirm its validity for CSF [31] and pleural and ascitic fluid [32]. Nevertheless, it is essential that systematic qualitative review of the differential scattergram (DIFF) and the HF-BF* count is performed to enable the development of algorithms that help determine whether microscopy review is indicated [33]. In this sense, Buoro et al. [32], [33] recommend microscopic review in the presence of WBC-BF cellularity ranges between 4.0 and 7.0/μL in CSF, and/or abnormal DIFF-scattergram, which may be caused by the presence of microorganisms and lead to misdiagnosis [34] or discrepancy between TNC-BF and WBC-BF count and the resulting increase of HF-BF* [31].

In synovial fluid, cell count in the BC-6800 analyzer can replace optical microscopy. Sample pre-treatment with hyaluronidase is recommended [35]. Although WBC-BF count in peritoneal dialysis fluid is transferable to count by optical microscopy, its transferability for differential counting has not been assessed [30].

To the best of our knowledge, no study has been published so far on the performance of this analyzer on pericardial fluid.

#### Unicel DxH (*Beckman Coulter*) series

Based on impedance-based Coulter technology, Blood Unicel DxH 800/900, analyzers incorporate a specific mode for TNC-BF and RBC-BF count in body fluids, a use that has been approved by the FDA. In addition, as research parameters, they provide differential nucleated cell count of two populations, PMN-BF and MN-BF. These analyzers also have specific quality control materials for this type of sample.

The recent study conducted by Cho et al. [21] confirmed the transferability of results from Unicel DxH 800, as compared to optical microscopy, for TNC-BF and RBC-BF in serous and synovial fluids. However, in CSF, only TNC-BF count was generalizable, but not RBC-BF, PMN-BF and MN-BF, with deviations >20% with respect to the gold-standard method. In addition, in this type of fluid, the limit of detection for TNC-BF was 18 cells/μL, which exceeds the upper limit of reference (5 cells/μL) recommended for microscopic review [7]. With respect to imprecision, within-run imprecision remained <10% for three levels of control. Table 1 details other analytical characteristics of this analyzer.

#### Cell-Dyn (*Abbott Diagnostics*) analyzers

Cell-Dyn analyzers, including CELL-DYN Saphire are hematology analyzers that combine impedance and optical microscopy for cell counting. Differential leukocyte count is performed by Multi Angle Polarized Scatter Separation (MAPSS). The main limitation of this analyzers is that it does not include a specific FDA-approved application for the analysis of body fluids. The volume of sample required is 120 μL.

Unlike other hematology analyzers, there is scarce literature available on the performance of Cell-Dyn analyzers for cell count in body fluids [25], [36], [37], [38], [39]. De Smet et al. [38] (Table 1) evaluated the precision of the Sapphire model in CSF, serous and peritoneal dialysis fluids and recommended reporting WBC-BF and RBC-BF count only if >50/μL and 3,000/μL, respectively. The reason is the significant imprecision of Sapphire at low cellularity, which exceeds 80% in leukocyte counts <5/μL, which limits its use in CSF. In addition, the same study demonstrated the lack of transferability in fluids with counts below the estimated limit of quantification. Moreover, the analyzer was unable to perform differential classification of leukocytes in serous fluids, probably due to classification of mesothelial cells and macrophages as polymorphonuclear cells. The lack of transferability was confirmed by Keuren et al. [39].

This analyzer features a flagging system for the detection of abnormal cells. A more recent study demonstrated the ability of Cell-Dyn Sapphire to detect tumor cells in serous fluids by cellular immunophenotyping [40].

Cell-Dyn analyzers will be replaced with the Alinity-hq analyzer, with a specific application for body fluids, which performance in cell count in blood was recently evaluated [41]. There is no data available about its performance in body fluids. However, recent internal evaluations demonstrate an enhanced cell detection and classification performance, and a reduction in the volume of sample required [42], [43].

#### GloCyte analyzer (*Advanced Instruments Inc.*)

The GloCyte analyzer, recently approved by the FDA, performs RBC-BF and TNC-BF count in CSF (Table 1). Unlike hematology analyzers, this method is based on the detection of the fluorescence of nucleated cells by nucleic acid staining. RBC count is performed using antibodies labelled with fluorochrome using a semi-conductor laser and an optic cell imaging system. The main advantages of this method is the small volume of sample required (30 μL), the short turnaround time (5 min), and the prevention of carryover through a cartridge system that prevents direct contact of the sample with the instrument. Furthermore, calibration is not necessary, and internal quality control materials are provided. The main limitation of this analyzer is that it has only been designed for CSF processing. In addition, differential nucleated cell count cannot be performed, and traditional manual counting is required in the presence of abnormal counts. This analyzer also lacks a flagging system for other types of cells, and has a narrow analytical measurement range.

The studies published so far have demonstrated the transferability of results for TNC-BF and RBC-BF [44], [45]. With respect to precision, this analyzer shows a coefficient of variation below 20% for counts of the two types of cells. This precision kept below 20% when control materials and patient specimens with counts near clinically-relevant cut-offs were analyzed [44].

### Urine analyzers

#### Sysmex analyzers

Sysmex analyzers (*Sysmex España, SL*) of the UF series are urine analyzers based on the analysis of particles by fluorescence flow cytometry. Thus, cells are classified according to three properties: size, internal complexity and nucleic acid content. The different versions of this analyzer (UF-1000i and UF-5000/4000) feature a specific cell count module for body fluids, although this application has not been approved by the FDA. According to the manufacturer, the limit of detection of the two analyzers is 2 leukocytes/μL and 5 RBCs/μL, respectively. Sample pre-treatment is not required, but the volume of sample required is 600 μL, which is one of its most relevant limitations, especially in the case of CSF. This system performs a rinse cycle to prevent carryover contamination. Internal quality control material is also provided. Evaluation studies show a between-run and within-run imprecision for the different types of cells of <10 and <20%, respectively, even at low cellularity [46], [47]. The characteristics of these analyzers described in the literature [21], [46], [47], [48] are shown in Table 2. Special attention needs to be paid to variability in the limits of quantification obtained in the different studies evaluating UF-5000 analyzers, probably due to methodological differences.

**Table 2:** Analytical characteristics of urine analyzers for the analysis of body fluids.

References	Fluids	LOB	LOD	LOQ	Carryover	Linearity
		(as cells/μL)				
UF-1000i analyzer (Sysmex)						
[46]	CSF	WBC: 0.1 RBC: 1.2	WBC: 0.7 RBC: 5.5	WBC: 2.4 RBC: 18.0	RBC: 0.00% WBC: < 0.13%	Tested in intervals: RBC: 1.9–970/μL (r=1.00) WBC: 0.8–405/μL (r=1.00) Difference between the average and the expected value ± 10%
[48]	Ascitic, pleural and peritoneal dialysis fluid	Not assessed	Not assessed	WBC: 9.2 RBC: 25.0	<0.01%	RBC: r^b^=0.99 WBC: r^b^=1 Intervals not specified

**UF-5000 analyzer (Sysmex)**						

[47]	CSF	TNC: 1 WBC: 1 RBC: 2	TNC: 1.8 WBC: 1.8 RBC: 3.5	TNC: 1.9 WBC: 1.9 RBC: 14	0.00%	Tested in intervals: RBC: 930–93,759/μL (r^b^=0.99) TNC: 3–2,957 (r^b^=0.99) WBC: 3–2,958/μL (r^b^=1)
[21]	CSF, ascitic, pleural, pericardial, synovial and peritoneal dialysis fluid	TNC: <1 RBC: <1	TNC: 2 RBC: <1	TNC: 25^a^ RBC: <1^a^	Not assessed	Not assessed

**Iris iQ2000 analyzer**						

[51]	CSF Pleural, ascitic and pericardial fluid Drain fluids	Not assessed	Not assessed	Not assessed	RBC: 0% TNC: 0%	RBC: max. 44,000/μL (r^b^ > 0.9) TNC: max. 2,600/μL (r^b^ > 0.9)
[52]	CSF, ascitic, pleural, pericardial and peritoneal fluid	Not assessed	Not assessed	TNC: 35^b^ RBC: 30^b^	Not assessed	Not assessed

LOB, limit of blank; LOD, limit of detection; LOQ, limit of quantification (minimum cell count that can be obtained with a coefficient of variation ≤20%). CSF, cerebrospinal fluid; TNC-BF, total cell count; WBC-BF, white blood cell count; PMN-BF, polymorphonuclear cell count; MN-BF, mononuclear cell count; RBC-BF, red blood cell count. ^a^LOQ estimated according to CSIF EP17-A2 protocol. ^b^Referred to as LOD in the manuscript.

The main difference between the two analyzers is based on the analytes assayed. The UF-1000i analyzer provides TNC-BF, WBC-BF and RBC-BF count, and a research parameter called “large cells”, which includes mesothelial cells, macrophages, and non-hematopoietic malignant cells [46]. This analyzer does not perform differential nucleated cell count. The UF-5000 analyzer performs TNC-BF, WBC-BF and differential count including mononuclear (MN-BF) and polymorphonuclear cells (PMN-BF) expressed as absolute values and percentages; RBC-BF, “*epithelial cells*” (EC), which include mesothelial cells and bacteria [47], which has been recently demonstrated to be useful for the detection and identification of bacteria as a predictor of a positive culture result [49]. One of the main limitations of this system is that it does not feature a flagging system indicating the need for review by optical microscopy.

A diversity of studies has evaluated the level of agreement between the two analyzers and the gold-standard method, the counting chamber. Fleming et al. [48] demonstrated a good level of agreement between RBC-BF count in UF-1000i and the counting chamber, which was acceptable for WBC-BF in specimens with counts <30 cells/μL. This is consistent with the results obtained by Buoro et al. [46] and more recently by Maleb et al. [50]. In these studies, the analyzer overestimated WBC-BF count. The software features an error flagging system that indicates the presence of interfering factors such as lipids, proteins, cell debris, bacteria, yeast, and incomplete red cell lysis, which aggregation leads to falsely elevated cell counts. Fleming et al. [48] describe falsely elevated counts associated with the unnoticed presence of yeast and bacteria. This interference is related to the method employed, flow cytometry, as the aggregation of these microorganisms, which contain small amounts of nucleic acids, would generate signals that mimic those of leukocytes and RBCs. Therefore, the authors recommend that scattergrams are routinely reviewed and, in the presence of alterations, optical microscopy is employed. Seghezzi et al. reported a good level of agreement for RBC-BF count between the UF-5000 analyzer and the gold-standard technique [47]. For TNC-BF and WBC-BF, the authors described a slight positive bias (10/μL for WBC-BF and 8.2/μL for TNC-BF), which decreased with counts <20/μL (1.8/μL for WBC-BF and 2.5/μL for TNC-BF). Nevertheless, this bias was not clinically significant. The recent study conducted by Cho et al. [21] reported a tendency to underestimation in leukocyte differential count (PMN-BF and MN-BF) on automated cell counters, as compared to manual counting.

#### Iris iQ200 analyzer (*Iris Diagnostics)*

IQ 200 series analyzers, marketed in Spain by *Beckman Coulter*, feature an FDA-approved mode that performs TNC-BF and RBC-BF count in body fluids, although differential nucleated cell count is not available. This analyzer is based on *Digital Flow Morphology* technology, which performs cell quantification and characterization. The Auto-Particle Recognition software generates and classifies individual cell images, which enables review and validation on the screen and the identification of cells with abnormal morphology. Unlike other analyzers, this equipment requires two previous dilutions of the sample, with a required volume of low cellularity specimens of 200 μL for serous fluids and 500 μL for CSF, which may be a limitation for the analysis of this type of sample [51], [52]. As other analyzers, Iris iQ200 features specific control materials for body fluids.

Studies on the transferability of results as compared to the gold-standard method show a good level of agreement between the two methods, even for TNC-BF <10 μL [51], [52], [53]. Reported imprecision values vary across studies according to specimen cellularity or the control materials employed for evaluation. Thus, Butch et al. [53] have reported a between-run and within-run imprecision of ≤10% for RBC-BF and TNC-BF, respectively. However, in other studies [51], [52], coefficients of variation exceeded 10% or were even higher than those obtained by manual counting [51]. The main analytical characteristics of the Iris iQ200 analyzer are shown in Table 2.

### Role of manual count of body fluids in the context of automated counting

Automated cell counters for body fluids are gradually replacing manual cell counting in routine laboratory practice [6]. However, automated cell counting in body fluids has some limitations. First, not all analyzers have an adequate analytical sensitivity for body fluids such as CSF [7]. The introduction of new technologies will contribute to overcome this limitation [54]. Secondly, although some analyzers provide differential counts for four populations, including neutrophils, lymphocytes, monocytes, and eosinophils [54], most are validated for PMN-BF and MN-BF only, which contravenes *International Council of Standardization in Haematology* (ICSH) recommendations. Indeed, the CLSI recommends the inclusion of all hematopoietic cells. In addition, the use of the term “mononuclear cell”, which includes lymphocytes, monocytes, immature granulocytes and blasts is not recommended [7]. This differentiation is very relevant in clinical settings such as malignant pleural effusion, where the neutrophil/lymphocyte ratio is used to establish patient prognosis [55]. Finally, automated counters for body fluids cannot recognize or report on other types of non-hematopoietic cells, including lining cells of different origins such as mesothelial cells, blasts, lymphoma cells, cells derived from solid tumors and atypical cells. This cellularity must always be included in the laboratory report, which must provide a morphologic description.

As mentioned above, some analyzers feature a flagging system called high-fluorescent cells (*HF-cells*). This system indicates the presence of cells with a high nucleus-cytoplasm ratio and a high nucleic acid content. Since the presence of HF-cells is associated with mesothelial and/or malignant cells, their detection has been proposed as an indication for microscopic review [33]. Routine use of HF-cells, however, has some limitations due to the lack of methodological standardization [33] and disagreement among scholars about the count or percentage of HF-cells that indicate the need for review by optical microscopy. Moreover, different primary goals and criteria have been used in studies defining a positive optical microscopy result, most of which were performed using Sysmex counters [17], [56], [57], [58], [59], [60], [61], [62], [63], [64], [65] (Table 3). HF-BF does not only contain neoplastic cells, but also benign cells such as macrophages and mesothelial cells, which reduces the specificity of this analyte for the detection of malignancy [66], with values ranging from 55 to 87% [61], [62], [63]. The specificity of the Sysmex-XN 1000 analyzer for the detection of HF-cells can be improved by adapting the algorithm to the characteristics of malignant cells [67]. Finally, some factors reduce the sensitivity of this analyte [61], [62], [63]. HF-BF count varies significantly according to the type of tumor, with higher values being found in carcinomas, as compared to lymphoproliferative diseases and mesothelioma, where malignant cells do not strictly meet the criteria for a cell to be classified as a HF-cell.

**Table 3:** Main studies assessing the performance of HF-cells.

References	Fluid	Analyzer	AUC ROC	HF-BF^a^ cut-off point	
[17]	CSF Ascitic, pleural and other fluids (bronchoalveolar lavage, peritoneal dialysis fluid, sinovial fluid, amniotic fluid, pericardial fluid, drains and cysts)	Sysmex XN-550	HF-BF%: 0.79 HF-BF#: 0.65	HF-BF%: 7.9/100 WBCs (S: 64.9%/Sp: 99.2%) HF-BF#: 46/μL (S: 49.2%/Sp: 82.0%)	
[56]	Ascitic fluid	Sysmex XN-1000	HF-BF%: 0.662 HF-BF#: 0.829	HF-BF%: 3.95/100 WBCs (S: 62.1%/Sp:58.9%) HF-BF#: >17/μL (S: 72.4%/Sp: 81.9%)	
	Pleural fluid		HF-BF%: 0.707 HF-BF#: 0.730	HF-BF%: 4.05/100 WBCs (S:62.5%/Sp: 71.3%) HF-BF#: >17/μL (S: 70.8%/Sp: 66.2%)	
	CSF		HF-BF%: 0.747 HF-BF#: 0.717	HF-BF%: 0.75/100 WBCs (S:66.7%/Sp: 79.3%) HF-BF#: >1/μL (S: 33.3%/Sp: 88.7%)	
[57]	Pleural fluid	Sysmex XN-9000	HF-BF%: 0.715 HF-BF#: 0.663 HF-BF% + CEA: 0.890 HF-BF# + CEA: 0.860	HF-BF%: 5.6/100 WBCs (S: 81.5%/Sp: 52.9%) HF-BF#: 29.5/μL (S: 70.4%/Sp: 61.8%) S: 96.3%/Sp: 73.5% S: 93.8%/Sp: 76.5%	
[58]	Pleural and ascitic fluid	Sysmex XN-9000	HF-BF%: 0.63 HF-BF#: 0.78	Not estimated HF-BF#: 68/μL (S: 61%/Sp: 100%)	
[59]	CSF^b^, ascitic, pleural, pericardial, peritoneal dialysis and synovial fluids and broncoalveolar lavage	Sysmex XN-9000	HF-BF%: 0.791 HF-BF#: Not estimated	HF-BF%: 6.9/100 WBCs (S: 87.2%/Sp: 60.4%) HF-BF#: Not estimated	
[60]	Ascitic fluid	Sysmex XN-350	TNC-BF#: 0.82 HF-BF%: 0.55 HF-BF#: 0.85	TCN-BF#: >341/μL HF-BF%: Not estimated HF-BF#: >28/μL Criterion for screening for peritoneal carcinomatosis: TC-BF# ≥250/μL and HF-BF# ≥17/μL (S: 91%/Sp: 77%)	
[61]	CSF Pleural, ascitic and pericardial fluid	Sysmex XN-1000	HF-BF%: 0.749 HF-BF#: 0.835	HF-BF%: 5.3/100 WBCs^c^ (S: 75%/Sp: 63%) HF-BF#: 67/μL^c^ (S: 73%/Sp: 87%)	
				HF-BF%: 2.7/100 WBCs^d^ (S: 92%/Sp: 40%) HF-BF#: 16/μL^d^ (S: 92%/Sp: 46%)	
[62]	Ascitic, pericardial, peritoneal dialysis and pleural fluid	Sysmex XN-2000	HF-BF%: 0.69 HF-BF#: 0.77	HF-BF%: 2.1/100 WBCs (S: 86%/Sp: 46%) HF-BF#: 17/μL (S: 88%/Sp: 61%)	
[63]	Pleural and ascitic fluid	Sysmex XN-1000	HF-BF%: 0.707 HF-BF#: 0.708	HF-BF%: 4.4/100 WBCs (S: 79.2/Sp: 55.8%) HF-BF#: 24.5/μL (S: 75.3%/55.0%)	
[64]	Pleural fluid	Sysmex XN-350	HF-BF# ≥17/μL (S: 94%/Sp: 50%) → AUC ROC: 0.718 HF-BF# ≥10/μL (S: 98%/Sp: 42%) → AUC ROC: 0.70 HF-BF# ≥17/μL (excluding patients with CHF and infection; S: 91%/Sp: 79%) → AUC ROC: 0.849 HF-BF# ≥10/μL (excluding patients with CHF and infection; S: 94%/Sp. 74%) → AUC ROC: 0.842 Recommended criterion for malignant pleural effusion screening: HF-BF# ≥17/μL		
[65]	Ascitic and pleural fluid	Sysmex XN-1000	Center 1: HF-BF# ≥108/μL → S: 66.7%/Sp: 93.6% HF-BF# ≥108/μL + clinical data → S: 100%/Sp: 68.9%		Center 2 HF-BF# ≥45/μL → S: 86.8%/Sp: 66.6% HF-BF# ≥45/μL + clinical data → S: 100%/Sp: Not estimated

CSF, cerebrospinal fluid; CHF, congestive heart failure; S, sensitivity; Sp, specificity; TNC-BF#, total nucleated cell count, expressed as absolute values; HF-BF%, total HF-cell count, expressed as percentages; HF-BF#, Total HF-cell count, expressed as absolute values. ^a^Recommended cut-off for reviewing cellularity by light microscopy or digital image analysis. ^b^On separate analysis of malignant CSF samples, the HF-BF of HF-cells did not show a significant correlation with the percentage of malignant cells by light microscopy. ^c^Selected cut-offs for maximizing sensitivity and specificity (Youden index). ^d^Selected cut-offs using sensitivity and negative predictive value as exclusion criteria

There is no data available on HF-BF* performance on the BC-6800 analyzer (Mindray) for the detection of malignant cells or as an indicator of microscopic review. Only a study, conducted by Buoro et al. [32], has been undertaken to assess the performance of this system in cell counting of serous fluids. The authors confirmed that all specimens with an abnormal scattergram showed a count >50 HF-cells/μL. Hence, the authors propose this as an indication of microscopic review.

A variety of algorithms have been proposed to determine the need for review by optical microscopy, some of which incorporate HF-cell and eosinophil count or other parameters related to lymphocyte morphology [17], [21], [58], [63], [64]. A recent study revealed that the sensitivity of HF-cell count improves when it is interpreted in combination with clinical data [64].

Concerning to urine analyzers, differences between total nucleated cell and white blood cell count and epithelial cell count can be used for indication of microscopic review, when using UF-5000 Sysmex. However, similar studies to those conducted in Sysmex hematology analyzers have not been found in the literature. A preliminary study recommends using the percentage count of mononuclear cells as an indicator of malignancy [68].

### Identification of cells in body fluid by digital imaging analysis

The use of automated morphological examination systems facilitates the analysis of blood samples. These systems use automated microscopy, digital image processing, and pattern recognition to identify and pre-classify the different types of normal blood cells [69]. One of the most widespread systems is CellaVision, which versions DM 96, DM9600, DM1200 and DI-60 are equipped with an application for body fluids. This application preclassifies nucleated cells as follows: neutrophils, eosinophils, lymphocytes, macrophages (including monocytes), other cells (basophils, lymphoma cells, blasts and tumor cells) and unidentified cells. This system examines cell morphology in body fluids, facilitates the detection of abnormal cells, and allows for consensual reevaluation of abnormal morphologies with other experts.

A diversity of studies has been conducted to assess the performance of the CellaVision system in assessing cell morphology in body fluids [70], [71]. Riedl et al. [70] used the DM96 version to compare the results obtained in different types of body fluids after cell classification by this system by optical microscopy. The authors demonstrated the transferability of results for the fluids included in the study. In addition, imprecision for the pre-classification of cells was <6% for all types of cells, and the percentage of cells that were correctly classified by the system in the pre-classification phase was 90 and 83% in CSF and other fluids, respectively. More recently, Takemura et al. [71] evaluated the mode for body fluids in the digital image analyzer DI-60 in CSF and serous fluids. Cell classification by this system showed a good correlation with the results obtained by optical microscopy for all populations except for monocytes, due to the morphological complexity of this type of cells.

## Conclusions

Automated cell counters are increasingly used for cell enumeration in body fluids in clinical laboratories. These analyzers have some advantages: most of them do not require a specimen pre-treatment; they reduce the laboratory turnaround time and exposure to biological risks; they require a low volume of sample; and are effective with low limits of detection and quantification [19].

However, these analyzers are complementary, not an alternative, to optical microscopy. In the future, automated counters will support cell classification by digital image analysis systems [20]. The incorporation of automated cell counters requires:

1. A deep understanding of their analytical properties, which must guarantee an adequate analytical sensitivity and imprecision for cell count in fluids with low cellularity. This is especially relevant to counts corresponding to cut-offs for clinical decision making used for fluid classification. Some authors [20] suggest that the “glass ceiling” has been broken, based on the results obtained with some automated counters [54].
2. Previous verification of system specifications following the ICSH protocol [6].
3. The use of systems equipped with a dedicated module for body fluids [4] that perform a rinse cycle to prevent carryover and cross-contamination by other specimens without requiring sample pre-treatment, except for synovial fluid due to its physical characteristics [11].
4. Evaluating imprecision in cell count for body fluids requires the availability of quality control materials for this type of specimen [6], [19].
5. For the analysis of body fluids, it is required to develop algorithms that incorporate decision-making criteria for the indication of microscopic review by and skilled specialist [2], [17], [58], [65].

There are still some challenges that hinder the spread of automated counters:

1. Further studies are required to assess the efficacy of flagging systems such as *HF-cells*, especially for screening malignant cells. At present, optical microscopy is the method of choice for cell counting in oncologic patients or on suspicion of a malignant effusion [56], [59], [61], [62], [63].
2. The *in vitro* diagnostics industry must focus their efforts on the development of technologies meeting CLSI recommendations for the evaluation of cell morphology in body fluids [2].
3. Specific quality specifications are required to assess the performance of automated analyzers in cell counting in body fluids [6].
4. Laboratories should engage in external quality control programs designed for these analytes [6].
5. There are no studies assessing the utility of automated cell count for amniotic fluid.
